# Mathematical models of the spread and consequences of the SARS-CoV-2 pandemics: Effects on health, society, industry, economics and technology

**DOI:** 10.1186/s13362-021-00111-w

**Published:** 2021-09-20

**Authors:** Alessandra Micheletti, Adérito Araújo, Neil Budko, Ana Carpio, Matthias Ehrhardt

**Affiliations:** 1 Università degli Studi di Milano, Milano, Italy; 2 University of Coimbra, Coimbra, Portugal; 3 Delft University of Technology, Delft, The Netherlands; 4 Universidad Complutense de Madrid, Madrid, Spain; 5 Bergische Universität Wuppertal, Wuppertal, Germany

## Editorial

The SARS coronavirus 2 (SARS-CoV-2) pandemic of coronavirus disease-19 (COVID-19) has changed the lives of everyone on the planet. As a new disease with a significant mortality rate and no known pharmaceutical intervention or curative treatment to date, COVID-19 has stimulated a huge worldwide academic research effort on every aspect of the spread of the virus and the effectiveness of containment measures, without neglecting the drawbacks that such measures have brought to the economy, society, and public health.

Within this framework, mathematicians and statisticians have contributed (and are actually still working on) models capable of capturing and focusing on the main components of epidemic spread and symptom severity, such as the dependence on patient age, the presence and role of asymptomatic infected individuals, the expected utilization of hospitals and intensive care units, the identification of the main sites and sources of infection (schools, restaurants, workplaces, etc.), the impact of lockdown measures.

In the highly heterogeneous scenario of COVID-19 epidemic spread, mathematical models adapted to national or more local situations are useful to provide policy makers with better insight into the consequences of different possible containment strategies, including the organization and optimization of vaccination campaigns.

For this reason, the European Consortium for Mathematics in Industry (ECMI) saw an urgency to provide policy makers with scientifically reliable studies on the many aspects of the problem, and this was our goal in producing this special issue of the Journal of Mathematics in Industry. This special issue is devoted to articles that propose data-driven mathematical and statistical models of the spread of the SARS-CoV-2 virus and/or its foreseeable consequences for public health, society, industry, business, and technology.

Actually, editorial activities such as these are only a first step toward the creation of a “scientific policy” that can truly counter the emergence of new health, environmental, and social emergencies. Such a scientific policy should act in a more systemic way, not just in the presence of emergencies. It should be the result of more rigorous collaboration between scientists from different disciplines (mathematicians, epidemiologists, virologists, environmental scientists, economists, social scientists, etc.) on the one hand, and between academia and science on the other: in contrast to the academic tendency to specialize and separate different fields of knowledge, policy makers should promote the “breaking of interdisciplinary silos” and realize an alliance between policy and science to more consciously address long-term policy strategic decisions.

The COVID-19 pandemic is just one example of the risks that humans face and that must be urgently addressed. The major challenge facing humanity in the coming years to prevent the rise of further emergencies is the environmental problem. As reported in the UNEP Frontiers 2016 Report [2], “climate change is a major factor for disease emergence. It influences the environmental conditions that can enable or disable the survival, reproduction, abundance, and distribution of pathogens, vectors, and hosts, as well as the means of disease transmission and the outbreak frequency. Growing evidence suggest that outbreaks of epidemic diseases may become more frequent as climate continues to change.”

Such problems can only be addressed with serious alignment of goals between scientists and policymakers, with the help and planning of good communication strategies, cf. [1].
